# An updated analysis of safety climate and downstream outcomes in two convenience samples of U.S. fire departments (FOCUS 1.0 and 2.0 survey waves)

**DOI:** 10.1186/s40621-024-00502-8

**Published:** 2024-05-21

**Authors:** Ashley M. Geczik, Jin Lee, Joseph A. Allen, Madison E. Raposa, Lucy F. Robinson, D. Alex Quistberg, Andrea L. Davis, Jennifer A. Taylor

**Affiliations:** 1 Department of Environmental and Occupational Health, Dornsife School of Public Health, Drexel University, Philadelphia, PA 19104 USA; 2 Department of Psychological Sciences, Kansas State University, Manhattan, KS USA; 3 Department of Family and Preventative Medicine, University of Utah, Salt Lake City, UT USA; 4 Department of Epidemiology and Biostatistics, Dornsife School of Public Health, Drexel University, Philadelphia, PA USA; 5 Dornsife School of Public Health, Urban Health Collaborative, Drexel University, Philadelphia, PA USA

**Keywords:** Safety climate, Descriptive epidemiology, Organizational outcomes, Safety outcomes, Injury prevention

## Abstract

**Background:**

The Fire service Organizational Culture of Safety (FOCUS) survey is an assessment tool comprised of psychometrically validated metrics of safety climate, safety behavior, and downstream outcomes (organizational and injury) that are specific to the U.S. fire and rescue service.

**Methods:**

This analysis consists of a descriptive summary of two independent survey waves (FOCUS 1.0 and 2.0). The fire departments included in these survey waves were from convenience sampling (*n*_1.0_ = 275; *n*_2.0_ = 170). In addition to department level characteristics, we examined individual level characteristics for firefighters and EMS providers in participating departments (*n*_1.0_ = 22,719; *n*_2.0_ = 16,882). We conducted regression analyses to examine the associations between safety climate and safety behaviors, organizational outcomes, and safety outcomes. All analyses were stratified by organization type (career, volunteer).

**Results:**

Our analysis indicated that a majority of respondents were males (90.7%_FOCUS 1.0_; 90.4%_FOCUS 2.0_), non-officers (68.4%_FOCUS 1.0_; 66.4%_FOCUS 2.0_), and non-Hispanic Whites (70.8%_FOCUS 1.0_; 69.5%_FOCUS 2.0_). For both samples there was a higher prevalence of injuries among individuals in career departments (*n*_FOCUS 1.0_ = 3778 [17.5%]; *n*_FOCUS 2.0_ = 3072 [18.7%]) than volunteer departments (*n*_FOCUS 1.0_ = 103 [8.8%]; *n*_FOCUS 2.0_ = 34 [7.4%]). We observed an approximate 10-point difference between the mean scores of Management Commitment to Safety for career and volunteer departments in both samples. We observed associations for two organizational outcomes, Safety Behavior and Job Satisfaction, with Management Commitment to Safety and Supervisor Support for Safety overall and when stratified by organization type. We observed a decrease in the odds of injuries associated with a one-unit increase in Management Commitment to Safety (OR_1.0 overall_: 0.98, 95% CI 0.97–0.99; OR_2.0 volunteer_: 0.90, 95% CI 0.85–0.95) and Supervisor Support for Safety (OR_1.0 overall_: 0.95, 95% CI 0.93–0.97; OR_1.0 career_: 0.95, 95% CI 0.92–0.98).

**Conclusions:**

From our current study, and a prior analysis of a geographically stratified random sample of U.S. fire departments, we identified that from all the organizational outcomes, job satisfaction was most consistently associated with FOCUS safety climate. Further, firefighters in our samples consistently rated Supervisor Support for Safety higher than Management Commitment to Safety. Future interventions should support fire departments in improving their departmental Management Commitment to Safety and maintaining their Supervisor for Safety.

**Supplementary Information:**

The online version contains supplementary material available at 10.1186/s40621-024-00502-8.

## Background

In 2020, it was estimated that 62.3 per 1000 firefighters were injured on the job (Campbell and Evarts 2020), underscoring the burden of injuries among this occupational group. Colloquial understanding of firefighter injuries may assume that most of their occupational injuries are related to fighting fires (i.e., on the fireground (Marsh, et al. 2018)), but prior research has demonstrated many injuries occur during training/physical activity exercises (Hollerbach et al. 2020; Poplin et al. 2012) or on non-fire calls (Poplin et al. 2012; Phelps et al. 2018). Emergency medical services (EMS) response constitutes 65% of all 9-1-1 calls to fire departments (Available from 2019) and there is some evidence to support that paramedics stationed in fire departments have higher all-injury rates than firefighters within the same department (Widman et al. 2018). Additionally, EMS providers have higher odds of experiencing injury from violence and assaults (Taylor et al. 2016; Maguire et al. 2018; Reichard et al. 2017).

Traditional firefighter and EMS provider injury research has primarily focused on the outcome of injuries (Marsh et al. 2018; Hollerbach et al. 2020; Poplin et al. 2012; Phelps et al. 2018; Available from 2019; Widman et al. 2018; Taylor et al. 2016; Maguire et al. 2018; Reichard et al. 2017), thus more needs to be known about its upstream predictors of safety climate, which is a well-established predictor of occupational injury (Christian et al. 2009; Huang et al. 2016). Safety climate reflects the perceptions of individuals regarding their organization’s regard for their occupational safety as expressed through their policies, procedures, and practices (Zohar 1980). An industry specific safety climate scale, the Fire service Organizational Culture of Safety (FOCUS) survey, was developed and its psychometric validation described previously (Taylor et al. 2019). The FOCUS survey is an assessment tool that is now in its fourth wave of assessment. Its research to practice impact has been described previously (Davis et al. 2020).

A previous analysis of the FOCUS beta-test survey wave identified two fire-service specific safety climate dimensions: Management Commitment to Safety and Supervisor Support for Safety (Taylor et al. 2019). FOCUS Management Commitment to Safety refers to members' perception that management (leadership) values their safety and engages in communication and actions that support safety procedures and practices (Taylor et al. 2019). FOCUS Supervisor Support for Safety refers to members' belief that their supervisors (company officers) value their safety based on communication, encouragement, and consequences (Taylor et al. 2019). A recent analysis of the FOCUS beta-test survey wave, a geographically stratified random sample, identified that the mean Management Commitment to Safety scores for career fire departments were lower compared to combination and volunteer departments, however a similar variation for mean Supervisor Support for Safety scores were not observed (Geczik et al. 2022). Safety behavior refers to the types of behaviors of workers that contribute to safety in the fire department, such as using the correct safety equipment (Neal and Griffin 2002). On the FOCUS survey we measured safety behavior using two metrics: Safety Behavior and Safety Compliance.

In the current study, we conducted a secondary analysis of cross-sectional survey data for two independent survey waves. The purpose of this study was to independently investigate the descriptive statistics of the individual and department level data from U.S. fire departments that completed the FOCUS 1.0 and 2.0 survey. Further, we were interested in examining the relationships between FOCUS safety climate and outcomes of interest (i.e. safety behaviors, organizational outcomes, and safety outcomes). This study replicates the published work of Geczik AM et al. (2022), using updated survey wave data (FOCUS 1.0 and 2.0) to further understand safety climate in a subset of U.S. career and volunteer fire departments. We wanted to replicate the previous analysis since there was a change in sampling methods from the FOCUS beta-test (random) to that used in FOCUS 1.0 and 2.0 survey waves (convenience).

## Methods

### Study population

The departments surveyed in the FOCUS 1.0 (2017–2018) and 2.0 (2019–2020) survey waves constituted convenience samples. Enrollment into these survey waves was open to any fire department in the U.S. that was interested in assessing their departmental safety climate to better understand their organizational safety culture on a first come, first serve basis until resources were exhausted. Survey administration was conducted over a 30-day period, on average, and departments had a goal to include at least 60% of members at each station. The FOCUS 1.0 survey wave included 31,508 individual respondents from 304 departments. The FOCUS 2.0 survey wave included 20,141 individual respondents from 178 departments. Both survey waves included U.S. fire departments that were career and volunteer and from urban, suburban, and rural areas.

### Demographic variables

Both FOCUS surveys obtained demographic information on the individual level. These variables have been previously described for the descriptive analysis of the FOCUS beta-test survey wave (Geczik et al. 2022). Respondents of the survey were asked to “select all that apply” when answering questions regarding their race/ethnicity, and professional role. The survey's response options for race and ethnicity were as follows: American Indian or Alaska Native, Asian, Black or African American, Hispanic, Native Hawaiian or Other Pacific Islander, Other, White. Individuals that self-identified as more than one race and ethnicity were categorized as “more than one.” Due to small numbers in our analytic sample and to preserve anonymity, our reported “Other” group included individuals that self-identified as: American Indian or Alaska Native, Asian, Native Hawaiian or Other Pacific Islander, or Other. For reporting race and ethnicity, we followed the 2021 guidelines from the Journal of the American Medical Association (JAMA) (Flanagin et al. 2021).

On the survey, the response options for “what is your role in this fire department” were as follows: firefighter, paramedic, emergency medical technician (EMT), Lieutenant, Captain, Battalion Chief (Division/District), or Chief (Fire, Deputy, Associate, Assistant), Commissioner. Since individuals could select more than one professional role, we created a three-level categorical variable (non-officer, officer, leadership) to identify the officer status of individual respondents based on the highest rank they reported. Individuals may hold officer or leadership roles in addition to being cross-trained firefighters and EMS providers (EMTs or Paramedics), single role-firefighters, or single-role EMS providers. In many municipalities, firefighters are cross-trained as they also respond to medical calls. The categorical variable was created based on the self-identification of rank: firefighter, EMT, or paramedic (non-officer); lieutenant or captain (officer); battalion chief, chief, or commissioner (leadership) (Geczik et al. 2022).

### Department characteristics

Department characteristics (i.e. call volume, population served, organization type, roster size) were collected during enrollment. Continuous variables were recategorized based on quantiles to create four categories (minimum to Q1, Q1 to median, median to Q3, Q3 to maximum). The State reported by each department was matched to its corresponding Federal Emergency Management Agency (FEMA) Region (*n* = 10). During enrollment, departments reported their Insurance Services Office (ISO) rating and Center for Public Safety Excellence (CPSE) accreditation, when applicable. ISO ratings are measures of a fire department’s ability to protect its community based on its preparedness to fight fires based upon relevant NFPA Standards (National Firefighter Protection Association et al. 2014). CPSE accreditation is a voluntary process that U.S. fire departments can undergo to ensure they provide the best support for their communities (Center for Public Safety Excellence 2024).

### Exclusion criteria

We restricted our analytic sample to departments that provided both fire and EMS response because we wanted to study the differences between those call types. Additional file 2: Supplemental Fig. 1 shows a flowchart of the exclusion criteria. Our exclusionary criteria for the analysis at the individual level was as follows: individuals missing fire department name on survey (*n*_FOCUS 1.0_ = 115; *n*_FOCUS 2.0_ = 82), individuals missing scale items of interest (*n*_FOCUS 1.0_ = 6,542; *n*_FOCUS 2.0_ = 2,869), individuals missing fire department identifier (*n*_FOCUS 1.0_ = 203), individuals under the age of 18 (*n*_FOCUS 1.0_ = 44; *n*_FOCUS 2.0_ = 49), individuals missing all demographics of interest (*n*_FOCUS 1.0_ = 435; *n*_FOCUS 2.0_ = 73), individuals from departments that did not fully complete enrollment (*n*_1.0_ = 133), individuals from departments with data entry errors (*n*_FOCUS 1.0_ = 411; *n*_FOCUS 2.0_ = 146), individuals from departments that did not provide EMS response (*n*_FOCUS 1.0_ = 853; *n*_FOCUS 2.0_ = 13), individuals over the age of 90 (*n*_FOCUS 1.0_ = 23; *n*_FOCUS 2.0_ = 22), and individuals with implausible years of experience (*n*_FOCUS 1.0_ = 30; n_FOCUS 2.0_ = 5).

At the department level, the following exclusions were made: departments that did not receive a fire department identifier due to incomplete survey (*n*_FOCUS 1.0_ = 2), departments that did not provide completed demographic data (*n*_FOCUS 1.0_ = 3), departments with data entry errors (*n*_FOCUS 1.0_ = 10; *n*_FOCUS 2.0_ = 3), departments that did not provide EMS response (*n*_FOCUS 1.0_ = 11; *n*_FOCUS 2.0_ = 5), and departments missing outcomes of interest (*n*_FOCUS 1.0_ = 3).

These exclusions resulted in the following analytic sample for each FOCUS survey wave: FOCUS 1.0 had 22,719 individuals from 275 departments and FOCUS 2.0 had 16,882 individuals from 170 departments. The analytic sample had an average response rate of 80.9% and 74.1%, for FOCUS 1.0 and 2.0 survey waves respectively.

### Safety climate metrics

For this analysis, the primary predictors of interest were the FOCUS safety climate scales Management Commitment to Safety and Supervisor Support for Safety (Taylor et al. 2019). Each of these constructs have 7-items with a 5-point Likert scale response. The psychometric properties of the measures, in terms of construct and criterion-related validities, have been previously reported (Taylor et al. 2019). Individual survey responses were aggregated to the group level (fire department) to calculate a departmental mean score (5-point scale) for Management Commitment to Safety and Supervisor Support for Safety, separately. The departmental means were converted to a 100-point scale for interpretability by the fire service and are presented as such in this paper.

### Outcome variables

We analyzed the following outcomes: safety behaviors (Safety Behavior, Safety Compliance), organizational outcomes (Engagement, Job Satisfaction), and injury outcomes (injury status in the past 12 months). Safety Behavior measures the safety awareness and behaviors among firefighters. Safety Compliance measures the degree to which firefighters act in accordance with established safety protocols, processes, and standards regarding fire runs (Taylor et al. 2019). Engagement measures the work-related state characterized by absorption and dedication to the job (Taylor et al. 2019). Engagement was assessed separately on EMS and fire service runs. Job Satisfaction measures the degree of positivity about work (Taylor et al. 2019). These four metrics were asked on a 5-point Likert scale, anchored with *Strongly Disagree* and *Strongly Agree*. The number of items per metric are as follows: 3-items (Safety Behavior); 4-items (Safety Compliance and Job Satisfaction); 6 items (Engagement). Individual survey responses were aggregated to the group level (fire department) to calculate a departmental mean score for each outcome. The departmental means were converted to a 100-point scale for interpretability by the fire service. The analyses presented in this paper use the converted scores on the 100-point scale. Respondents were asked about their injury status, “During the past 12 months were you injured while performing your job?”, with the following response options: yes, no. Respondents were asked, “Did that injury require medical treatment or consultation?”, with response options of yes or no. We cleaned this variable to report back responses for only those that indicated they were injured in the past 12-months.

### Statistical analysis

We reported descriptive statistics of all continuous (mean ± standard deviation (SD), range) and categorical (counts, percentages) variables from both analytic samples. In addition to overall summaries, we stratified by organization type (career, volunteer) to evaluate any potential differences related to organizational structure of the participating fire departments. Pearson correlation matrices were computed to investigate correlations between continuous measures at the department and individual levels.

*Organizational Outcomes:* Multivariable linear regression models were used to estimate the relationship between organizational outcomes and FOCUS safety climate, adjusting for departmental characteristics. Individual models were run for each organizational outcome and FOCUS safety climate predictor (Management Commitment to Safety and Supervisor Support for Safety). The linear regression models were adjusted for roster size, annual call volume, and population served. These models were run for the entire sample of departments and stratified by organization type. For models stratified by organization type, the adjustment variables were recategorized based on quartiles for each corresponding organization type due to inherent differences in size for career versus volunteer departments.

*Individual Outcomes:* Given the clustered nature of the data, multilevel logistic regression models were used to estimate the odds of self-reported injury from the past 12 months with FOCUS safety climate mean scores (Management Commitment to Safety, Supervisor Support for Safety). For our analysis, we analyzed both survey waves independently from one another. Further, in each wave, individuals were nested within departments for a multilevel approach. These models were run for each predictor for the total sample and then stratified by organization type. We calculated estimated associations using generalized estimated equation (GEE) methods to account for potential correlation between individuals within a department. Individual identifiers were not collected for respondents, thus linkage across survey respondents could not be taken into account. Effects were summarized using odds ratios (OR) and 95% confidence intervals (CI). These models were adjusted for age, years of experience, sex (male, female), and officer status (non-officer, officer, leadership). To conduct a complete case analysis for the logistic regression model we made the following additional exclusions: individuals missing injury status (*n*_FOCUS 1.0_ = 353; *n*_FOCUS 2.0_ = 127), age (*n*_FOCUS 1.0_ = 1028; *n*_FOCUS 2.0_ = 816), years of experience (*n*_FOCUS 1.0_ = 1327; *n*_FOCUS 2.0_ = 400), sex (*n*_FOCUS 1.0_ = 828; *n*_FOCUS 2.0_ = 528), and officer status (*n*_FOCUS 1.0_ = 218; *n*_FOCUS 2.0_ = 199). The final analytic sample for the logistic regression models were 17,627 individuals and 275 departments for FOCUS 1.0 wave and 14,806 individuals and 170 departments for FOCUS 2.0 wave. The two FOCUS waves were analyzed separately for this analysis.

Sensitivity analyses included: (1) Organization type as a covariate in the linear and logistic regression models as an alternative to stratification to increase power, due to small sample size of volunteer departments, and assess if the regression results are similar; (2) Exclude departments that have assessed their safety climate using the FOCUS survey more than once as their department may be more attune of their overall safety; (3) Exclude departments that did not achieve 60% response rate from their survey response (*n*_FOCUS 1.0_ = 60; *n*_FOCUS 2.0_ = 33); and (4) Compare the FOCUS 2.0 descriptive statistics among departments that finished assessing between September 1, 2019 and March 1, 2020 (*n* = 82) and those that finished assessing between April 1, 2020 and July 1, 2020 (*n* = 88), due to the impact of COVID-19 response among a subset of U.S. fire departments (Raposa et al. 2023).

Statistical significance was set to < 0.05 for all analyses. All statistical analyses were conducted using SAS 9.4 (Cary, North Carolina). The protocol received Institutional Review Board approval from Drexel University.

## Results

### Descriptive statistics

The individual-level demographics and characteristics were similar between the FOCUS 1.0 and 2.0 survey waves (Table 1). In the FOCUS 1.0 analytic sample there were 22,719 respondents (*n*_1.0 career_ = 21,551 [94.9%]; *n*_1.0 volunteer_ = 1168 [5.1%]) and in the FOCUS 2.0 sample there were 16,882 respondents (*n*_2.0 career_ = 16,421 (97.2%); n_2.0 volunteer_ = 461(2.8%)). Overall, the majority of respondents were males (*n*_FOCUS 1.0_ = 20,611 [90.7%]; *n*_FOCUS 2.0_ = 15,258 [90.4%]), non-officers (*n*_FOCUS 1.0_ = 15,548 [68.4%]; *n*_FOCUS 2.0_ = 11,206 [66.4%]), and non-Hispanic Whites (*n*_FOCUS 1.0_ = 16,086 [70.8%]; *n*_FOCUS 2.0_ = 11,733 [69.5%]). For both samples there was a higher prevalence of injuries in career departments (*n*_FOCUS 1.0_ = 3778 [17.5%]; *n*_FOCUS 2.0_ = 3072 [18.7%]) than volunteer departments (*n*_FOCUS 1.0_ = 103 [8.8%]; *n*_FOCUS 2.0_ = 34 [7.4%]). Among those that were injured 68.7% (FOCUS 1.0) and 65.9% (FOCUS 2.0) required medical treatment following the injury.

**Table 1:** Descriptive characteristics of the FOCUS 1.0 and 2.0 analytic samples stratified by fire department organization type

	FOCUS 1.0						FOCUS 2.0					
	Total population		Career department		Volunteer department		Total population		Career department		Volunteer department	
	*n* = 22,719		*n* = 21,551		*n* = 1,168		*n* = 16,882		*n* = 16,421		*n* = 461	
Individual level characteristics	mean ± SD	min–max	mean ± SD	min–max	mean ± SD	min–max	mean ± SD	min–max	mean ± SD	min–max	mean ± SD	min–max
Age	40.4 ± 9.8	18.0–88.0	40.5 ± 9.6	18.0–88.0	38.1 ± 12.9	18.0–79.0	40.8 ± 9.87	18.0–88.0	40.9 ± 9.75	18.0–88.0	39.1 ± 13.2	18.0–75.0
Years of experience	16.5 ± 9.5	1.00–56.0	16.6 ± 9.3	1.00–56.0	15.2 ± 12.1	1.00–56.0	15.9 ± 9.55	1.00–60.0	15.9 ± 9.46	1.00–58.0	13.6 ± 12.1	1.00–60.0
	*n*	%	*n*	%	*n*	%	*n*	%	*n*	%	*n*	%
Sex												
Male	20,611	90.7	19,615	91.0	996	85.3	15,258	90.4	14,863	90.5	395	85.7
Female	1068	4.7	965	4.5	103	8.8	1061	6.3	1012	6.2	49	10.6
Missing	1040	4.6	971	4.5	69	5.9	563	3.3	546	3.3	17	3.7
Rank												
Firefighter	4198	18.5	3798	17.6	400	34.2	3121	18.5	2999	18.3	122	26.5
Paramedic	456	2.0	449	2.1	7	0.6	335	2.0	333	2.0	< 5	-
EMT	186	0.8	159	0.7	27	2.3	176	1.0	164	1.0	12	2.6
Lieutenant	1735	7.6	1682	7.8	53	4.5	1291	7.6	1269	7.7	22	4.8
Captain	1958	8.6	1912	8.9	46	3.9	1488	8.8	1467	8.9	21	4.6
Battalion Chief	754	3.3	704	3.3	50	4.3	515	3.1	508	3.1	7	1.5
Chief/Commissioner	242	1.1	193	0.9	49	4.2	221	1.3	201	1.2	20	4.3
More than one selected	12,812	56.4	12,300	57.1	512	43.8	9375	55.5	9127	55.6	248	53.8
Missing	378	1.7	354	1.6	24	2.1	360	2.1	353	2.1	7	1.5
Officer status*												
Non-officer	15,548	68.4	14,633	67.9	815	69.8	11,206	66.4	10,883	66.3	323	70.1
Officer	5684	25.0	5486	25.5	198	17.0	4411	26.1	4331	26.4	80	17.4
Leadership	1209	5.3	1078	5.0	131	11.2	905	5.4	854	5.2	51	11.1
Missing	378	1.7	354	1.6	24	2.1	360	2.1	353	2.1	7	1.5
Race and Ethnicity												
Hispanic	1623	7.1	1607	7.5	16	1.4	1282	7.6	1273	7.8	9	2.0
More than one	1235	5.4	1214	5.6	21	1.8	917	5.4	905	5.5	12	2.6
Non-Hispanic Black or African American	1157	5.1	1145	5.3	12	1.0	1233	7.3	1227	7.5	6	1.3
Non-Hispanic White	16,086	70.8	15,032	69.8	1054	90.2	11,733	69.5	11,324	69.0	409	88.7
Other**	1539	6.8	1515	7.0	24	2.1	992	5.9	976	5.9	16	3.5
Missing	1079	4.7	1038	4.8	41	3.5	725	4.3	716	4.4	9	2.0
Education												
Less than high school	38	0.2	35	0.2	< 5		30	0.2	28	0.2	< 5	-
High school or equivalent	8984	39.5	8475	39.3	509	43.6	6502	38.5	6334	38.6	168	36.4
Undergraduate degree	11,950	52.6	11,445	53.1	505	43.2	9174	54.3	8945	54.5	229	49.7
Graduate degree	1060	4.7	967	4.5	93	8.0	892	5.3	839	5.1	53	11.5
Missing	687	3.0	629	2.9	58	5.0	284	1.7	275	1.7	9	2.0
Injury last 12 months												
No	18,485	81.4	17,436	80.9	1049	89.8	13,649	80.8	13,231	80.6	418	90.7
Yes	3881	17.1	3778	17.5	103	8.8	3106	18.4	3072	18.7	34	7.4
Missing	353	1.6	337	1.6	16	1.4	127	0.8	118	0.7	9	2.0
Medical Treatment for Injury^¥^												
No	1203	31.0	1171	31.0	32	31.1	1053	33.9	1041	33.9	12	35.3
Yes	2666	68.7	2595	68.7	71	68.9	2047	65.9	2025	65.9	22	64.7
Missing	12	0.3	12	0.3	0	0.0	6	0.2	6	0.2	0	0.0

	Total departments		Career department		Volunteer department		Total departments		Career department		Volunteer department	
	*n* = 275		*n* = 206		*n* = 69		*n* = 170		*n* = 145		*n* = 25	
Fire department characteristics	mean ± SD	min–max	mean ± SD	min–max	mean ± SD	min–max	mean ± SD	min–max	mean ± SD	min–max	mean ± SD	min–max
Percent EMS runs	67.7 ± 18.7	1.00–95.0	70.5 ± 15.2	1.00–95.0	59.5 ± 24.9	1.00–95.0	65.5 ± 18.7	2.36–96.7	68.0 ± 15.1	8.00–96.7	51.1 ± 28.9	2.36–86.7
Percent fire runs	25.9 ± 19.8	1.00–99.0	22.1 ± 15.8	1.00–88.0	37.0 ± 25.7	1.00–99.0	26.4 ± 21.5	1.00–97.6	23.5 ± 18.5	1.00–92.0	43.6 ± 29.2	2.00–97.6
Engagement on EMS runs	73.8 ± 5.30	61.3–93.3	72.7 ± 4.86	61.3–90.0	77.1 ± 5.27	65.2–93.3	73.9 ± 4.84	63.1–88.5	73.0 ± 4.23	63.1–82.3	78.8 ± 5.25	69.3–88.5
Engagement on fire runs	82.2 ± 3.80	72.7–93.3	81.7 ± 3.35	72.7–92.2	83.6 ± 4.63	73.6–93.3	83.8 ± 3.86	73.0–93.6	83.5 ± 3.57	73.0–93.6	85.2 ± 5.07	73.3–93.3
Job Satisfaction	78.2 ± 7.69	56.7–92.5	77.1 ± 7.50	56.7–92.5	81.3 ± 7.40	59.6–92.0	78.1 ± 7.14	61.1–98.3	77.0 ± 6.63	61.1–92.7	84.7 ± 6.49	69.2–98.3
Safety Behavior	86.0 ± 5.98	66.2–97.8	85.8 ± 5.52	66.2–96.7	86.6 ± 7.21	68.7–97.8	86.6 ± 6.02	68.6–98.9	86.5 ± 5.94	69.3–97.3	87.4 ± 6.53	68.6–98.9
Safety Compliance	86.4 ± 5.03	64.5–98.3	87.2 ± 4.38	64.5–98.3	84.3 ± 6.17	67.0–93.3	88.3 ± 4.74	63.3–96.2	88.9 ± 3.98	78.9–96.2	84.8 ± 6.96	63.3–92.9
Management Commitment	71.4 ± 9.30	46.0–94.6	68.5 ± 8.14	46.0–90.7	79.9 ± 7.20	57.4–94.6	70.0 ± 10.0	45.3–98.6	67.8 ± 8.67	45.3–88.7	82.9 ± 7.21	61.5–98.6
Supervisor Support	81.2 ± 4.34	63.1–97.0	80.8 ± 3.67	69.2–90.4	82.1 ± 5.83	63.1–97.0	83.0 ± 3.88	71.8–98.1	82.6 ± 3.36	71.8–93.1	85.6 ± 5.46	74.9–98.1
	*n*	%	*n*	%	*n*	%	*n*	%	*n*	%	*n*	%
*Roster size*												
0–24	40	14.5	28	13.6	12	17.4	18	10.6	13	9.0	5	20.0
25–49	85	30.9	57	27.7	28	40.6	39	22.9	29	20.0	10	40.0
50–99	78	28.4	54	26.2	24	34.8	53	31.2	46	31.7	7	28.0
100 +	72	26.2	67	32.5	5	7.2	60	35.3	57	39.3	3	12.0
Missing	0	0.0	0	0.0	0	0.0	0	0.0	0	0.0	0	0.0
*Annual number of calls*												
0–499	16	5.8	0	0.0	16	23.2	11	6.5	3	2.1	8	32.0
500–999	26	9.5	4	1.9	22	31.9	11	6.5	4	2.8	7	28.0
1000–4999	120	43.6	91	44.2	29	42.0	61	35.9	53	36.6	8	32.0
5000–9999	42	15.3	40	19.4	2	2.9	36	21.2	34	23.4	2	8.0
10,000+	70	25.5	70	34.0	0	0.0	51	30.0	51	35.2	0	0.0
Missing	1	0.4	1	0.5	0	0.0	0	0.0	0	0.0	0	0.0
*Population served*												
0–4999	17	6.2	1	0.5	16	23.2	5	2.9	1	0.7	4	16.0
5000– 9999	21	7.6	7	3.4	14	20.3	13	7.6	7	4.8	6	24.0
10,000–24,999	67	24.4	42	20.4	25	36.2	37	21.8	28	19.3	9	36.0
25,000–49,999	67	24.4	56	27.2	11	15.9	36	21.2	31	21.4	5	20.0
50,000–99,999	43	15.6	42	20.4	1	1.4	32	18.8	31	21.4	1	4.0
100,000+	53	19.3	53	25.7	0	0.0	45	26.5	45	31.0	0	0.0
Missing	7	2.5	5	2.4	2	2.9	2	1.2	2	1.4	0	0.0
*FEMA region*												
1	22	8.0	12	5.8	10	14.5	13	7.6	10	6.9	3	12.0
2	15	5.5	6	2.9	9	13.0	8	4.7	5	3.4	3	12.0
3	22	8.0	15	7.3	7	10.1	14	8.2	10	6.9	4	16.0
4	37	13.5	32	15.5	5	7.2	26	15.3	25	17.2	1	4.0
5	70	25.5	50	24.3	20	29.0	38	22.4	30	20.7	8	32.0
6	19	6.9	18	8.7	1	1.4	11	6.5	10	6.9	1	4.0
7	23	8.4	17	8.3	6	8.7	12	7.1	11	7.6	1	4.0
8	17	6.2	15	7.3	2	2.9	12	7.1	10	6.9	2	8.0
9	24	8.7	23	11.2	1	1.4	23	13.5	23	15.9	0	0.0
10	26	9.5	18	8.7	8	11.6	13	7.6	11	7.6	2	8.0
*CPSE accrediation*												
No	244	88.7	176	85.4	68	98.6	145	85.3	121	83.4	24	96.0
Yes	31	11.3	30	14.6	1	1.4	25	14.7	24	16.6	1	4.0
*ISO rating*												
High (1, 2, 3)	161	58.5	147	71.4	14	20.3	127	74.7	116	80.0	11	44.0
Medium (4, 5, 6)	93	33.8	48	23.3	45	65.2	33	19.4	24	16.6	9	36.0
Low (7, 8, 9, 10)	8	2.9	2	1.0	6	8.7	2	1.2	0	0.0	2	8.0
Missing	13	4.7	9	4.4	4	5.8	8	4.7	5	3.4	3	12.0

*If more than one rank was selected, the highest level of rank was designated for this categorization

**Due to small sample sizes in some categories, Other includes individuals that self-identified as: Asian, Native Hawaiian or Pacific Islander, American Indian or Alaskan Native, and Other

^¥^This variable was calculated from only those that reported an injury, thus the n's will not add up to the total individuals in the category and the percentage is based on those that were injured

**Fig. 1:**
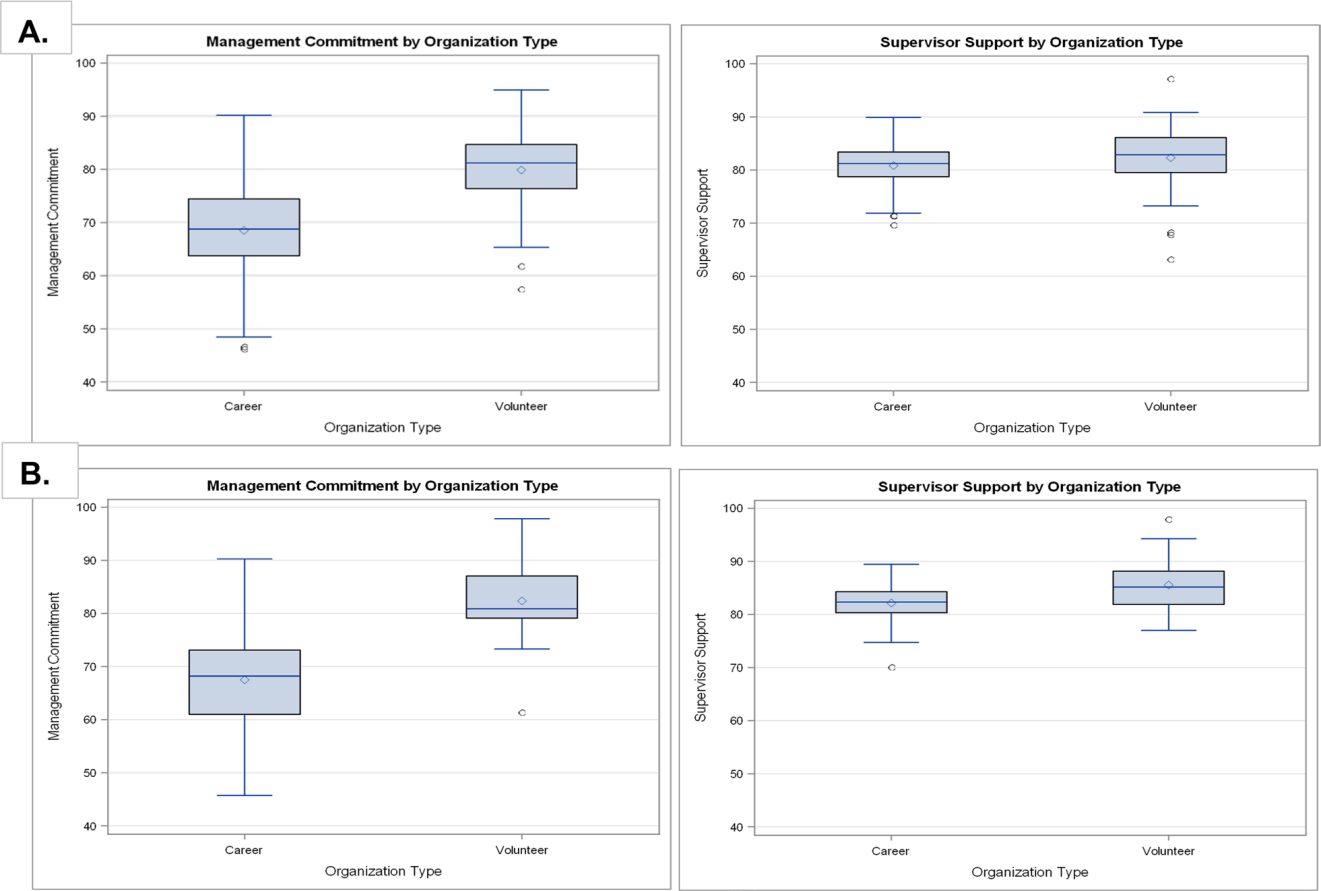
Box and whisker plots comparing mean FOCUS safety climate scores by organization type. **A** Comparison between mean safety climate scores (Management Commitment to Safety and Supervisor Support for Safety) by organization type for the FOCUS 1.0 survey wave. **B** Comparison between mean safety climate scores (Management Commitment to Safety and Supervisor Support for Safety) by organization type for the FOCUS 2.0 survey wave

For the FOCUS 1.0 analytic sample there were 275 departments (*n*_1.0 career_ = 206 (74.9%); *n*_1.0 volunteer_ = 69 (25.1%)) and for the FOCUS 2.0 analytic sample there were 170 departments (*n*_2.0 career_ = 145 (85.3%); n_2.0 volunteer_ = 25 (14.7%)). Most departments were career departments (74.9% in FOCUS 1.0 and 85.3% in FOCUS 2.0). The mean scores, standard deviations (SD), and ranges for safety climate at the department level are reported (Table 1). For the total population, we observed approximately a 10-point difference in the mean scores for Engagement on EMS calls (mean_FOCUS 1.0_ = 73.8; mean_FOCUS 2.0_ = 73.9) versus Engagement on fire calls (mean_FOCUS 1.0_ = 82.2; mean_FOCUS 2.0_ = 83.8). We observed a similar difference of approximately 10-points among career departments for Engagement on EMS calls (mean_FOCUS 1.0_ = 72.7; mean_FOCUS 2.0_ = 73.0) versus Engagement on fire calls (mean_FOCUS 1.0_ = 81.7; mean_FOCUS 2.0_ = 83.5). Whereas the mean scores for volunteer departments were closer to 6-point difference for Engagement on EMS calls (mean_FOCUS 1.0_ = 77.1; mean_FOCUS 2.0_ = 78.8) versus Engagement on fire calls (mean_FOCUS 1.0_ = 83.6; mean_FOCUS 2.0_ = 85.2). The mean scores of Job Satisfaction (mean_FOCUS 1.0_ = 78.2; mean_FOCUS 2.0_ = 78.1), Safety Behavior (mean_FOCUS 1.0_ = 86.0; mean_FOCUS 2.0_ = 86.6), and Safety Compliance (mean_FOCUS 1.0_ = 86.4; mean_FOCUS 2.0_ = 88.3) were fairly similar between samples, which held when stratified by organization type. We observed an increase in mean scores for Management Commitment to Safety when stratified by organization type, with volunteer departments having a higher mean score than career departments in both sample waves (Table 1; Fig. 1). For FOCUS 1.0, we observed an 11-point difference in the mean Management Commitment to Safety scores between career and volunteer departments, with volunteer departments having the highest mean score. For FOCUS 2.0, we observed a 15-point difference between career and volunteer departments, with volunteer departments having the highest mean score. A similar increase in mean scores by organization type was not observed for Supervisor Support for Safety. Box plots of the FOCUS safety climate metrics by department size variables (roster size, annual call volume, and population served) appear in Additional file 2: Fig. 2A and B. For both FOCUS waves, we observed more variation in the mean Management Commitment to Safety scores with the different size variables compared to the mean Supervisor Support for Safety scores. We observed that larger departments had lower mean Management Commitment to Safety scores compared to smaller departments.

There was variation in fire departments participation across the FEMA regions (Table 1). FEMA region 5 (Illinois, Indiana, Michigan, Minnesota, Ohio, and Wisconsin) had the most participation (25.5%_FOCUS 1.0_; 22.4%_FOCUS 2.0_). The FEMA region that had the least participation (5.5%_FOCUS 1.0_; 4.7%_FOCUS 2.0_) was FEMA region 2 (New Jersey and New York).

The Pearson correlation matrices are visualized at the department (Table 2) and individual level (Table 3). At the department level, we observed moderate positive correlations between annual call volume with roster size [*r*_FOCUS 1.0_ = 0.58; *r*_FOCUS 2.0_ = 0.88]. and with population served [*r*_FOCUS 1.0_ = 0.69; *r*_FOCUS 2.0_ = 0.58]. At the department level for both samples, we observed moderate positive correlations between Job Satisfaction and FOCUS safety climate (Management Commitment to Safety [*r*_FOCUS 1.0_ = 0.67; *r*_FOCUS 2.0_ = 0.69] and Supervisor Support for Safety [*r*_FOCUS1.0_ = 0.62; *r*_FOCUS2.0_ = 0.54]). For both survey waves, we observed moderate positive correlations between Safety Behavior and Safety Compliance [*r*_FOCUS1.0_ = 0.59; *r*_FOCUS2.0_ = 0.61].

**Table 2:** Department level Pearson correlation coefficient matrices for FOCUS 1.0 and FOCUS 2.0

	1.Percent fire runs	2.Percent EMS runs	3.Annual call volume	4.Roster size	5.Population served	6.Engagement—EMS	7.Engagement—fire	8.Job Satisfaction	9.Safety Behavior	10.Safety Compliance	11.Management Commitment	12.Supervisor Support
*FOCUS 1.0 (n = 269 departments)*												
1.Percent fire runs	1.00											
2.Percent EMS runs	**− 0.71**	1.00										
3.Annual call volume	− 0.11	0.15	1.00									
4.Roster size	− 0.14	0.11	**0.58**	1.00								
5.Population served	− 0.10	0.12	**0.69**	**0.73**	1.00							
6.Engagement on an EMS runs	0.17	− 0.07	− 0.19	− 0.18	− 0.20	1.00						
7.Engagement on a fire runs	0.14	− 0.22	− 0.05	− 0.05	− 0.05	**0.61**	1.00					
8.Job Satisfaction	0.14	− 0.09	0.03	0.03	0.07	0.41	0.47	1.00				
9.Safety Behavior	0.06	− 0.06	− 0.25	− 0.19	− 0.24	0.28	0.21	0.32	1.00			
10.Safety Compliance	− 0.12	0.04	− 0.14	0.03	− 0.08	0.15	0.11	0.03	**0.59**	1.00		
11.Management Commitment	0.28	− 0.24	− 0.34	− 0.32	− 0.31	**0.51**	0.41	**0.67**	0.48	0.01	1.00	
12.Supervisor Support	0.04	− 0.12	0.03	0.06	0.06	0.37	**0.53**	**0.62**	0.43	0.22	0.49	1.00
*FOCUS 2.0 (n = 168 departments)*												
1.Percent fire runs	1.00											
2.Percent EMS runs	**− 0.73**	1.00										
3.Annual call volume	− 0.10	0.12	1.00									
4.Roster size	− 0.11	0.13	**0.88**	1.00								
5.Population served	− 0.14	0.06	**0.58**	**0.57**	1.00							
6.Engagement on an EMS runs	0.23	− 0.06	− 0.16	− 0.16	− 0.13	1.00						
7.Engagement on a fire runs	0.11	− 0.25	− 0.06	− 0.09	− 0.03	0.41	1.00					
8.Job Satisfaction	0.13	− 0.16	0.002	− 0.01	0.003	0.32	0.32	1.00				
9.Safety Behavior	0.07	− 0.15	− 0.17	− 0.25	− 0.07	− 0.03	0.08	0.33	1.00			
10.Safety Compliance	− 0.09	0.06	− 0.02	− 0.09	0.02	− 0.08	0.11	0.004	**0.61**	1.00		
11.Management Commitment	0.25	− 0.31	− 0.30	− 0.33	− 0.17	0.37	0.23	**0.69**	0.44	− 0.11	1.00	
12.Supervisor Support	0.11	− 0.19	− 0.07	− 0.10	0.02	0.21	0.33	**0.54**	0.40	0.16	0.43	1.00

Bolded values indicate moderate (0.50–0.69) and high (0.70–0.99) correlations

**Table 3:** Individual level Pearson correlation coefficient matrices for FOCUS 1.0 and FOCUS 2.0

	1. Age	2. Years of experience	3. Engagement—EMS	4. Engagement—fire	5. Job Satisfaction	6. Safety Behavior	7. Safety Compliance	8. Management Commitment	9. Supervisor Support
*FOCUS 1.0 (n = 19,645 individuals)*									
1. Age	1.00								
2. Years of experience	**0.80**	1.00							
3. Engagement on an EMS runs	− 0.07	− 0.06	1.00						
4. Engagement on a fire runs	− 0.06	− 0.10	0.41	1.00					
5. Job Satisfaction	0.04	− 0.03	0.28	0.46	1.00				
6. Safety Behavior	− 0.08	0.01	0.44	0.02	0.03	1.00			
7. Safety Compliance	− 0.05	0.02	0.39	− 0.10	− 0.19	**0.73**	1.00		
8. Management Commitment	− 0.09	− 0.03	**0.50**	0.32	0.41	**0.68**	0.36	1.00	
9. Supervisor Support	− 0.04	− 0.08	0.33	**0.56**	0.43	0.21	0.13	0.31	1.00
*FOCUS 2.0 (n = 15,112 individuals)*									
1. Age	1.00								
2. Years of experience	**0.74**	1.00							
3. Engagement on an EMS runs	− 0.16	− 0.22	1.00						
4. Engagement on a fire runs	− 0.04	− 0.06	0.41	1.00					
5. Job Satisfaction	− 0.03	− 0.08	0.47	**0.51**	1.00				
6. Safety Behavior	0.08	− 0.06	− 0.14	0.22	0.37	1.00			
7. Safety Compliance	0.06	− 0.01	− 0.08	0.35	0.22	**0.69**	1.00		
8. Management Commitment	0.05	− 0.07	0.20	0.38	**0.68**	**0.61**	0.18	1.00	
9. Supervisor Support	0.05	− 0.08	0.29	0.39	**0.67**	**0.55**	0.43	**0.72**	1.00

Bolded values indicate moderate (0.50–0.69) and high (0.70–0.99) correlations

For individual-level characteristics, the notable correlations were not consistent between two samples (Table 3). For both samples, we observed a moderate positive correlation between Safety Behavior and Management Commitment for Safety [*r*_FOCUS1.0_ = 0.68; *r*_FOCUS2.0_ = 0.61]. For the FOCUS 2.0 survey wave, we observed a moderate positive correlation between Job Satisfaction and FOCUS safety climate (Management Commitment to Safety [*r*_FOCUS 2.0_ = 0.68] and Supervisor Support for Safety [*r*_FOCUS 2.0_ = 0.67]).

### Department-level organizational outcomes

In FOCUS 1.0, we observed that all outcomes were positively associated with both FOCUS Management Commitment to Safety and Supervisor Support for Safety when we did not stratify by organization type (Table 4). For FOCUS 2.0, we observed that all outcomes, except for Safety Compliance, were positively associated with Management Commitment to Safety. Further, we observed that all outcomes, except for Engagement on EMS runs, were positively associated with Supervisor Support for Safety.

**Table 4:** Linear regression models examining the relationship between safety climate scores and safety behaviors/organizational outcomes for FOCUS 1.0 and FOCUS 2.0 overall and stratified by organization type

	FOCUS 1.0 (*n* = 269 departments)						FOCUS 2.0 (*n* = 170 departments)					
	Overall						Overall					
	Management Commitment^a^			Supervisor Support^a^			Management Commitment^e^			Supervisor Support^e^		
	*n* = 269			*n* = 269			*n* = 170			*n* = 170		
	B	95% CI	*p*-value	B	95% CI	*p*-value	B	95% CI	*p*-value	B	95% CI	*p*-value
*Safety behaviors*												
Safety Behavior	0.39	(0.31, 0.47)	** < 0.001**	0.67	(0.51, 0.82)	** < 0.001**	0.35	(0.24, 0.46)	** < 0.001**	0.58	(0.36, 0.80)	** < 0.001**
Safety Compliance	0.09	(0.01, 0.16)	**0.03**	0.31	(0.17, 0.46)	** < 0.001**	0.04	(− 0.06, 0.13)	0.46	0.23	(0.04, 0.42)	**0.02**
*Organizational outcomes*												
Engagement on EMS Runs	0.22	(0.15, 0.29)	** < 0.001**	0.47	(0.34, 0.59)	** < 0.001**	0.11	(0.02, 0.20)	**0.02**	0.19	(− 0.01, 0.39)	0.06
Engagement on Fire Runs	0.16	(0.10, 0.21)	** < 0.001**	0.44	(0.35, 0.53)	** < 0.001**	0.09	(0.02, 0.17)	**0.02**	0.34	(0.19, 0.49)	** < 0.001**
Job Satisfaction	0.69	(0.61, 0.77)	** < 0.001**	1.07	(0.89, 1.25)	** < 0.001**	0.62	(0.52, 0.71)	** < 0.001**	0.88	(0.64, 1.11)	** < 0.001**

	Management Commitment to Safety						Management Ccommitment to Safety					
	Career Department^b^			Volunteer Department^d^			Career Department^f^			Volunteer Department^g^		
	*n* = 201			*n* = 68			*n* = 145			*n* = 25		
	B	95% CI	*p*-value	B	95% CI	*p*-value	B	95% CI	*p*-value	B	95% CI	*p*-value
*Safety behaviors*												
Safety Behavior	0.41	(0.32, 0.49)	** < 0.001**	0.53	(0.30, 0.77)	** < 0.001**	0.38	(0.27, 0.49)	** < 0.001**	0.81	(0.35, 1.27)	** < 0.01**
Safety Compliance	0.13	(0.05, 0.20)	**0.002**	0.20	(− 0.02, 0.42)	0.08	0.08	(− 0.005, 0.16)	0.07	0.34	(− 0.34, 1.02)	0.31
*Organizational outcomes*												
Engagement on EMS Runs	0.21	(0.13, 0.29)	** < 0.001**	0.24	(0.06, 0.41)	**0.01**	0.08	(− 0.01, 0.17)	0.07	0.01	(− 0.35, 0.37)	0.95
Engagement on fire runs	0.13	(0.07, 0.19)	** < 0.001**	0.27	(0.13, 0.41)	** < 0.001**	0.07	(-0.01, 0.15)	0.09	0.29	− -0.15, 0.72)	0.18
Job Satisfaction	0.68	(0.58, 0.78)	** < 0.001**	0.80	(0.65, 0.95)	** < 0.001**	0.60	(0.50, 0.70)	** < 0.001**	0.52	(0.12, 0.91)	**0.01**

	Supervisor Support for Safety						Supervisor Support for Safety					
	Career Department^b^			Volunteer Ddepartment^d^			Career Department^f^			Volunteer Department^g^		
	*n* = 201			*n* = 68			*n* = 145			*n* = 25		
	B	95% CI	*p*-value	B	95% CI	*p*-value	B	95% CI	*p*-value	B	95% CI	*p*-value
*Safety behaviors*												
Safety Behavior	0.59	(0.38, 0.79)	** < 0.001**	0.78	(0.51, 1.05)	** < 0.001**	0.67	(0.40, 0.95)	** < 0.001**	0.88	(0.15, 1.61)	**0.02**
Safety Compliance	0.42	(0.25, 0.58)	** < 0.001**	0.26	(-0.02, 0.54)	0.07	0.29	(0.10, 0.48)	** < 0.01**	0.58	(-0.32, 1.48)	0.19
*Organizational outcomes*												
Engagement on EMS runs	0.55	(0.38, 0.72)	** < 0.001**	0.28	(0.05, 0.50)	**0.02**	0.17	(-0.04, 0.38)	0.11	-0.04	(-0.53, 0.45)	0.85
Engagement on fire runs	0.52	(0.41, 0.63)	** < 0.001**	0.38	(0.21, 0.55)	** < 0.001**	0.34	(0.16, 0.51)	** < 0.001**	0.33	(-0.27, 0.93)	0.26
Job Satisfaction	1.08	(0.82, 1.34)	** < 0.001**	1.00	(0.81, 1.19)	** < 0.001**	0.92	(0.61, 1.22)	** < 0.001**	0.59	(-0.02, 1.16)	**0.04**

a. Adjusted for: roster size (0–31, 32–53, 54–104, 105–1556), annual call volume (35–1549, 1550–3999, 4000–10011, 10012–533594), population served (300–14399, 14400–31355, 31356–77145, 77146–2700000)

b. Adjusted for roster size (0–31, 32–58, 59–120, 121–1556), annual call volume (550–3228, 3229–5592, 5593–12155, 12156–522594) and population served (4500–24999, 25000–45763, 45764–109999, 110000–2700000)

d. Adjusted for roster size (11–29, 30–44, 45–69, 70–177), annual call volume (35–499, 500–849, 850–1399, 1400–6500) and population served (300–5149, 5150–9999, 10000–17999, 18000–75000)

e. Adjusted for: roster size (8–36, 37–69, 70–131, 132–3565), annual call volume (37–2137, 2138–5089, 5090–12996, 12997–452826), population served (800–17256, 17257–35999, 36000–104591, 104592–7555754)

f. Adjusted for roster size (8-43, 44-74, 75-177, 178-3565), annual call volume (274-2871, 2872-5999. 6000-15808, 15809-452826) and population served (800-23999, 24000-52254, 52255-119999, 120000-7555754)

g. Adjusted for roster size (15-29, 30-42, 43-54, 55-117), annual call volume (37-399, 400-901, 902-1499, 1500-6363) and population served (1250-5999, 6000-9999, 10000-20999, 21000-63914)

n's do not add up to 275 due to missing values (*n* = 6) for adjusted covariates (*n* = 5 for career and *n* = 1 for volunteer)

Bolded values are statistically significant at an alpha level of 0.05

B represents the unstandardized parameter estimate of beta

Overall, and stratified by organization type, we observed positive associations for Safety Behavior and Job Satisfaction with both FOCUS safety climate metrics. The estimated change in Safety Behavior was associated with a one-unit increase in mean Management Commitment to Safety (B_FOCUS 1.0 overall_: 0.39, 95% CI 0.31–0.47; B_FOCUS 2.0 overall_: 0.35, 95% CI 0.24–0.46). The estimated change in Safety Behavior was associated with a one-unit increase in Supervisor Support for Safety (B_FOCUS 1.0 overall_: 0.67, 95% CI 0.51–0.82; B_FOCUS 2.0 overall_: 0.58, 95% CI 0.36–0.80). The estimated change in Job Satisfaction was associated with a one-unit increase in mean Management Commitment to Safety (B_FOCUS 1.0 overall_: 0.69, 95% CI 0.61–0.77; B_FOCUS 2.0 overall_: 0.62, 95% CI 0.52–0.71). The estimated change in Job Satisfaction was associated with a one-unit increase in Supervisor Support for Safety (B_FOCUS 1.0 overall_: 1.07, 95% CI 0.89–1.25; B_FOCUS 2.0 overall_: 0.88, 95% CI 0.64–1.11). Consistently the magnitude of estimated change was strongest for Job Satisfaction, as indicated by a higher beta estimate. The results stratified by organization type are presented in Table 4. For the FOCUS 2.0 survey wave, results for volunteer departments should be interpreted cautiously due to smaller sample sizes.

### Individual-level safety outcomes

We reported the results of the unadjusted and adjusted multilevel logistic regression analyses in Table 5. In the adjusted models for FOCUS 1.0, we observed a 2% decrease in the odds of injury reported in the last 12 months associated with a one-unit increase in mean Management Commitment to Safety scores (OR_FOCUS1.0 overall_: 0.98, 95% CI 0.97–0.99). Additionally, we observed a 5% decrease in the odds of injury reported in the last 12 months associated with a one-unit increase in mean Supervisor Support for Safety scores (OR_FOCUS 1.0 overall_: 0.95, 95% CI 0.93–0.97; OR_FOCUS 1.0 career_: 0.95, 95% CI 0.92–0.98).

In the adjusted models for FOCUS 2.0, we observed an association between in the odds of injury reported the last 12 months and mean Management Commitment to Safety scores (OR_FOCUS 2.0 volunteer_: 0.90, 95% CI 0.85–0.95).

**Table 5:** Multilevel logistic regression between safety climate and injury status among individuals stratified by organization type for FOCUS 1.0 and 2.0

	FOCUS 1.0								
	All individuals (*n* = 17,627), All departments (*n* = 275)			Individuals (*n* = 16,703), Career departments (*n* = 206)			Individuals (*n* = 924), Volunteer departments (*n* = 69)		
	Estimate	95% CI	OR (95% CI)	Estimate	95% CI	OR (95% CI)	Estimate	95% CI	OR (95% CI)
*Unadjusted*									
Management Commitment	− 0.02	(− 0.03, − 0.01)	**0.98 (0.97, 0.99)**	− 0.01	(− 0.03, − 0.002)	0.99 (0.97, 1.00)	− 0.04	(− 0.09, 0.01)	0.96 (0.91, 1.01)
Supervisor Support	− 0.05	(− 0.07, − 0.03)	**0.95 (0.93, 0.97)**	− 0.05	(− 0.08, − 0.03)	**0.95 (0.92, 0.97)**	− 0.04	(− 0.09, 0.02)	0.96 (0.91, 1.02)
Adjusted*									
Management Commitment	− 0.02	(− 0.03, − 0.01)	**0.98 (0.97, 0.99)**	− 0.01	(− 0.02, − 0.001)	0.99 (0.98, 1.00)	− 0.04	(− 0.08, 0.01)	0.96 (0.92, 1.01)
Supervisor Support	− 0.05	(− 0.07, − 0.03)	**0.95 (0.93, 0.97)**	− 0.05	(− 0.08, -0.02)	**0.95 (0.92, 0.98)**	− 0.03	(-0.09, 0.02)	0.97 (0.91, 1.02)

	FOCUS 2.0								
	All individuals (*n* = 14,806), All departments (*n* = 170)			Individuals (*n* = 14,403), Career departments (*n* = 145)			Individuals (*n* = 403), Volunteer departments (*n* = 25)		
	Estimate	95% CI	OR (95% CI)	Estimate	95% CI	OR (95% CI)	Estimate	95% CI	OR (95% CI)
*Unadjusted*									
Management Commitment	− 0.02	(− 0.03, − 0.01)	**0.98 (0.97, 0.99)**	− 0.01	(− 0.02, 0.01)	0.99 (0.98, 1.01)	− 0.11	(− 0.16, − 0.05)	**0.90 (0.85, 0.95)**
Supervisor Support	− 0.01	(− 0.04, 0.02)	0.99 (0.96, 1.02)	− 0.001	(− 0.03, 0.03)	1.00 (0.97, 1.03)	− 0.09	(− 0.27, 0.09)	0.91 (0.76, 1.09)
Adjusted*									
Management Commitment	− 0.02	(− 0.03, − 0.004)	0.98 (0.97, 1.00)	− 0.01	(− 0.02, 0.01)	0.99 (0.98, 1.01)	− 0.10	(− 0.16, − 0.05)	**0.90 (0.85, 0.95)**
Supervisor Support	− 0.01	(− 0.04, 0.02)	0.99 (0.96, 1.02)	0.002	(− 0.03, 0.03)	1.00 (0.97, 1.03)	− 0.07	(− 0.25, 0.11)	0.93 (0.78, 1.12)

*Adjusted for age, years of experience, sex (male, female), and rank (non-officer, officer, leadership)

### Sensitivity analyses

We conducted sensitivity analyses to assess the robustness of our findings across various scenarios (Additional file 1: Tables 1–9). Notably, when organization type was considered as a factor, we no longer observed an association between the odds of injuries and Management Commitment to Safety in the FOCUS 1.0 survey wave sample (Additional file 1: Table 2). When we excluded the departments that assessed with FOCUS more than once, most of our associations held (Additional file 1: Tables 3–4). We did observe that the association between Safety Behavior and FOCUS safety climate as well as Job Satisfaction and FOCUS safety climate was null for volunteer departments in the FOCUS 2.0, which is likely due to the reduced sample size (Additional file 1: Table 3).

When we excluded departments that did not achieve a 60% response rate based on their reported roster size and number of departmental respondents (Additional file 1: Tables 5–6) we no longer observed an association between Engagement (on fire or EMS runs) and Management Commitment to Safety for volunteer departments (Additional file 1: Table 5), however the magnitudes of the associations were similar for Engagement on EMS runs and there was overlap in the 95% CI for Engagement on fire runs. The associations between Safety Behavior and FOCUS safety climate as well as with Job Satisfaction and FOCUS safety climate were null for volunteer departments. From the logistic regression analysis, compared to the main analysis, we no longer observed associations between the odds of injury and Supervisor Support for Safety among individuals in career departments for the FOCUS 1.0 survey wave (Additional file 1: Table 6). However, there was overlap in the 95% CI. The results for the volunteers should be interpreted cautiously due to reduced sample size for both survey waves.

In the FOCUS 2.0 data, we examined descriptive characteristics and regression analyses for departments that finished assessing before (September 1, 2019–March 1, 2020) and during (April 1, 2020–July 1, 2020) the U.S. response to the COVID-19 pandemic (Additional file 1: Tables 7–9). Descriptive characteristics were largely similar between both groups. Notably, those that finished assessing before March 1, 2020, reported a 16.5% injury rate in the last 12 months, compared to 19.4% for those that finished assessing after March 1, 2020. Linear regression results indicated associations between Engagement on EMS runs and Management Commitment to Safety (B: 0.17, 95% CI 0.04–0.29; *p*-value: 0.01), as well as between Engagement on fire runs and Management Commitment to Safety (B: 0.15, 95% 0.04–0.26; *p*-value: 0.01) for career departments who finished assessing before March 1, 2020, which were not observed in the main analysis or for those who finished assessing after March 1, 2020 (Additional file 1: Table 8). Further, in comparison to the main analyses the associations between Management Commitment to Safety and Supervisor Support for Safety with Job Satisfaction no longer held for volunteer departments for either assessment period. Logistic regression results showed a 2% decrease in the odds of injuries with a one-unit increase in Management Commitment to Safety among individuals who finished assessing before March 1, 2020, which was not observed in the overall FOCUS 2.0 sample (held (Additional file 1 Table 9). Further, we no longer observed an association with the decrease in odds of injuries for a one-unit increase in Management Commitment to Safety among volunteers that finished assessing before March 1, 2020.

## Discussion

The purpose of this study was to investigate the descriptive statistics of respondents to the FOCUS 1.0 and the FOCUS 2.0 survey waves, to better understand if two independent convenience samples were reflective of the geographically stratified random sample (FOCUS beta-test). A prior analysis of FOCUS beta-test data observed a dose response relationship when examining mean FOCUS Management Commitment to Safety by organization type (career, combination, and volunteer) (Geczik et al. 2022). Mean scores were lower among career departments compared to combination departments and to volunteer departments, however there was no difference in the mean scores of Supervisor Support for Safety (Geczik et al. 2022). While this current study only captured organization type on two levels, we still observed that career departments had a lower mean score of Management Commitment to Safety compared to volunteer departments. This further supports our previous claim that department size does matter, especially regarding member perceptions of their leadership’s response to their safety. Furthermore, Christian et al., found that management commitment to safety was a stronger driver of safety climate compared to supervisor support (Christian et al. 2009), so departments should focus on improving that score. Given discussions with our fire service partners about the structures of their organizations, the limited face time that leadership has with their members may be driving this phenomenon, especially in larger departments. We consistently observed lower mean Management Commitment to Safety scores compared to mean Supervisor Support for Safety scores using the data in this analysis and in the previously published analysis of the FOCUS beta-test data (Geczik et al. 2022). The lower mean Management Commitment to Safety scores are indicative of fire department members not necessarily hearing from their upper management leadership regarding their safety. If fire departments implement more face time between department leadership and members at the individual stations, the perceptions to Management Commitment to Safety may increase thus further reducing the odds of injuries. There are inherent organizational differences between career and volunteer fire departments, such as number of calls, size of population served, and work hours, that may also be affecting the differences in these scores.

A novel component of the initial analysis of the FOCUS beta-test data was to stratify by organization type, which was also assessed for the FOCUS 1.0 and 2.0 survey waves, however there were differences in the stratified groups sample sizes. In our current study we did observe that FOCUS 1.0 had a larger overall sample size compared to FOCUS 2.0 and FOCUS beta-test, which made it a more robust sample of career and volunteer departments. The FOCUS 2.0 sample had more career departments (*n* = 170) and a similar number of volunteer departments (*n* = 25) compared to the FOCUS beta-test (n_career_ = 125; n_volunteer_ = 24). We observed similar demographic characteristics between the individual respondents from the FOCUS 1.0 and FOCUS 2.0 survey waves, which is reassuring for comparing across the two convenience samples. Of the organizational outcomes analyzed in our study, Job Satisfaction was the most robust in terms of its relationship to FOCUS safety climate, which was consistent with the beta-test data. For this analysis we were able to examine the relationship between Safety Behavior and FOCUS safety climate because it was asked on both survey waves. We observed an association overall and by organization type for this downstream outcome as well. For our current analysis, this outcome was also associated with FOCUS safety climate for both samples. The reduction in the odds of injuries we observed for FOCUS safety climate among career departments in the FOCUS beta-test sample (Geczik et al. 2022) and FOCUS 1.0 sample was not observed in the FOCUS 2.0 sample. Further, unique to the FOCUS 2.0 sample, we observed a reduction in the odds of injuries associated with Management Commitment to Safety among volunteer departments. This association was not observed for volunteer departments in FOCUS 1.0 or the FOCUS beta-test (Geczik et al. 2022).

Based on the observed differences in the data, it would appear that the FOCUS 1.0 and 2.0 samples are not truly reflective of the FOCUS beta-test sample. This is likely due to the fact that the samples are from different departments overall, which would be expected given our understanding that safety climate is unique to the organization that is being examined. It is possible that the different sampling methods, convenience sampling versus random sampling, are contributing to these differences. Due to the higher number of departments that assessed in FOCUS 1.0 compared to FOCUS 2.0 and our previously studied FOCUS beta-test sample, we are unable to definitively conclude that the convenience sample of departments are reflective of the geographically stratified random sample.

### Study strengths and limitations

A strength of our current analysis was the large sample size we had of individual respondents across both survey waves, which provided us with more data to analyze the perceptions to the different metrics assessed in the FOCUS survey. Our study had some important limitations. Considerations should be made regarding the FOCUS 2.0 sample given the impact of COVID-19 on the U.S. fire service. As evidenced by our sensitivity analysis, there are some associations that are only present among the sample that completed assessing prior to March 1, 2020. Previous research has identified the impact of the COVID-19 pandemic on a sample of U.S. fire departments (Raposa et al. 2023). The researchers identified that there were concerns regarding the “low and decreasing scores” of Management Commitment to Safety in the sample (Raposa et al. 2023), which could explain why we observed associations in the regression analyses for Management Commitment to Safety among the departments that completed assessment prior to March 1, 2020. Additionally, the researchers identified that Engagement on EMS runs and Engagement on fire runs had an average score decrease over their 6-month study period (Raposa et al. 2023). Again, this may explain why we did not observe an association between these metrics in the overall sample, but did for the departments that assessed before March 1, 2020.

Further, given the voluntary participation for members within participating departments, there may be non-response bias present in our study samples. At the department level, there may be the presence of selection bias, as departments that are more in tune with their safety may be inclined to enroll to assess. Additionally, the data used for this study is self-reported by individual respondents, which may result in social desirability bias. Based on our previous work, we are not overly concerned with the presence of non-response bias, selection bias, or social desirability bias due to the heterogeneity of responses for each of the metrics (ranges in terms of mean scores). There is potential that nondifferential misclassification by outcome may arise, due to the self-reporting of injury among respondents due to recall bias. The outcome will likely be underreported in instances where minor injuries in the last year were forgotten. If present, we anticipate that this would bias the results towards the null. Additionally, the wording on the survey for the injury status question is open to interpretation by the respondent. The survey question does not account for varying levels of severity and does not ask about the number of injuries an individual experienced in a year, so we are not able to ascertain the full burden of injuries among our survey population.

There is potential that aggregation fallacy is present for our logistic regression results as we are attributing the departmental mean scores of Management Commitment to Safety and Supervisor Support for Safety to the individuals within departments. This was done because FOCUS safety climate constructs are meant to be measured at the group level. If present, this may bias our results toward the null. Finally, while we accounted for the department and individual level clustering of this data, we were unable to account for station level hierarchical clustering due to data quality issues for the station level identifiers that were inconsistently self-reported by individuals and their departmental contact. Previously, Supervisor Support for Safety has been identified as a station level construct (Taylor et al. 2019), however in our current analysis we could only assess it on the departmental level. However, our findings of positive relationships with Supervisor Support for Safety and odds of injuries at the department level indicate these relationships might be stronger when assessed at the station level. Future survey iterations should create numeric only options for station identifiers to prevent data management limitations in classifying station level identifiers.

Additionally, the stratification by organization type (career, combination, volunteer) turned out to be an important variable, so it should be added back into future waves of FOCUS. While we consider our large sample a strength of this study, we must mention that the small number of volunteer departments in the FOCUS 2.0 survey wave presented some concerns regarding power. Future iterations of FOCUS should aim to recruit a larger sample of volunteer and combination departments to further support the stratification of these constructs by organization type.

## Conclusion

This evaluation of the descriptive statistics of two independent convenience samples helps inform our understanding of safety climate within the sample of U.S. fire department that have taken the FOCUS survey. The findings from our current analysis suggest that the FOCUS 1.0 and 2.0 survey waves are not reflective of the geographically stratified random sample from the FOCUS beta-test survey wave. However, both of these samples are larger, in terms of individual respondents, than the initial FOCUS wave which highlights the reach of the FOCUS assessment tool.

Our current research confirms previous findings that the size of a fire department does have an impact on the organizational safety climate, however additional analyses of convenience samples with the three levels of organization types represented is warranted. Further, our regression analyses show that both Management Commitment to Safety and Supervisor Support for Safety are important upstream factors in the maintenance of positive organizational outcomes and reduction of firefighter injury. Future research should investigate potential interventions to help our fire service partners increase these scores, with a particular focus on Management Commitment to Safety. Interventions can aim to support departments in maintaining their Supervisor Support for Safety scores.

### Supplementary Information

**Additional file 1**:** Supplemental Table 1.** Linear regression models examining the relationship between safety climate scores and safety behaviors/organizational outcomes for FOCUS 1.0 and FOCUS 2.0, adjusting for organization type.** Supplemental Table 2.** Multilevel logistic regression between safety climate and injury status for FOCUS 1.0 and 2.0, adjusting for organization type.** Supplemental Table 3.** Linear regression models examining the relationship between safety climate scores and safety behaviors/organizational outcomes for FOCUS 1.0 and FOCUS 2.0 overall and stratified by organization type, excluding departments that have assessed with FOCUS more than once.** Supplemental Table 4.** Multilevel logistic regression between safety climate and injury status among individuals stratified by organization type for FOCUS 1.0 and 2.0, excluding departments that have assessed with FOCUS more than once.** Supplemental Table 5.** Linear regression models examining the relationship between safety climate scores and safety behaviors/organizational outcomes for FOCUS 1.0 and FOCUS 2.0 overall and stratified by organization type, excluding departments with <60% response rate.** Supplemental Table 6.** Multilevel logistic regression between safety climate and injury status among individuals stratified by organization type for FOCUS 1.0 and 2.0, excluding departments with <60% response rate.** Supplemental Table 7.** Descriptive characteristics of FOCUS 1.0 and 2.0 analytic sample stratified by fire department organization type, for comparison between departments that assessed before and during the U.S. COVID-19 response.** Supplemental Table 8.** Linear regression models examining the relationship between safety climate scores and safety behaviors/organizational outcomes for FOCUS 2.0 overall and stratified by organization type, for comparison between departments that assessed before and during the U.S. COVID-19 response.** Supplemental Table 9.** Multilevel logistic regression between safety climate and injury status among individuals stratified by organization type for FOCUS 2.0, for comparison between departments that assessed before and during the U.S. COVID-19 response.

**Additional file 2: Supplemental Figure 1.** Flowcharts of the analytic samples for FOCUS 1.0 and FOCUS 2.0 survey waves.** Supplemental Figure 2A.** Box and whisker plots comparing FOCUS safety climate scores by size variables for FOCUSv.1.0 departments.** Supplemental Figure 2B.** Box and whisker plots comparing FOCUS safety climate scores by size variables for FOCUSv.2.0 departments.

## Data Availability

The datasets generated and analyzed for this current study are not publicly available due to conditions of the IRB. The data are available from the corresponding author upon reasonable request.
